# Visiting Scholars Program to enhance career development among early-career KL2 investigators in Clinical and Translational Science: Implications from a quality improvement assessment

**DOI:** 10.1017/cts.2020.564

**Published:** 2020-12-14

**Authors:** Sheri L. Robb, Thomas H. Kelly, Victoria L. King, Jason T. Blackard, Patricia C. McGuire

**Affiliations:** 1 School of Nursing, Indiana University, Indianapolis, IN, USA; 2 College of Nursing, University of Kentucky, Lexington, KY, USA; 3 College of Medicine, University of Kentucky, Lexington, KY, USA; 4 College of Medicine, University of Cincinnati, Cincinnati, OH, USA; 5 Indiana Clinical and Translational Sciences Institute, Indianapolis, IN, USA

**Keywords:** Research education, career development, clinical transnational sciences

## Abstract

CTSI Career Development Award (KL2) programs provide junior faculty with protected time and multidisciplinary, mentored research training in clinical and translational science research. The *KL2 Visiting Scholars Program* was developed to promote collaborative cross-CTSA training, leverage academic strengths at host CTSAs, and support the career development of participating scholars through experiential training and the development of new partnerships. This manuscript provides a detailed programmatic description and reports outcomes from post-visit and outcomes surveys. Since 2016, 12 scholars have completed the program, with 6 scheduled to complete it in 2021. Post-visit surveys (*n* = 12) indicate all scholars reported the program valuable to career development, 11 reported benefit for research development, and 11 expansion of collaborative networks. Outcomes surveys (*n* = 11) revealed subsequent scholar interaction with host institution faculty for 10 scholars, 2 collaborative grant submissions (1 funded), 2 planned grant submissions, 1 published collaborative manuscript, and 3 planned manuscript/abstract submissions. The Visiting Scholars Program is a cost- and time-efficient program that leverages the academic strengths of CTSAs. The program enhanced KL2 scholar training by expanding their professional portfolio, promoting research development, and expanding collaborative networks. Resources to support the program are shared in this report to expedite the development of similar programs at regional and national levels.

## Introduction

The National Center for Advancing Translational Sciences (NCATS) through its Clinical and Translational Science Awards (CTSA) program supports a national network of medical research institutions (called *hubs*) that are working collaboratively to accelerate innovations in research methods, training, and career development [1]. An important feature of every CTSA program hub is the Institutional Career Development Award (KL2) which provides junior faculty with protected time and multidisciplinary, mentored research training in clinical and translational science research [2]. The overarching goal of the KL2 program is to support the development of early-career scientists and their success as lead investigators. Essential to each scholar’s success are opportunities to present and discuss their work with senior scientists, form productive cross-institutional collaborations, and expand their professional networks.

Faculty engagement in clinical service, education, and research is the cornerstone of academic health centers and is facilitated by faculty development opportunities at their home institutions [3–7]. Senior faculty are frequently invited to give presentations at other institutions, provide plenary sessions at conferences, serve on grant review boards, and assume leadership roles within professional organizations, but junior faculty have fewer opportunities. Faculty exchange programs, particularly those designed with junior faculty in mind, provide junior faculty with the opportunity to meet established researchers with similar interests outside of their own universities, develop new research collaborations, engage in joint publications, and increase their professional networks and visibility [6–8].

Junior faculty can enhance career development by broadening their exposure to educational and research resources at other CTSA institutions. Compared with extended externships that may require several weeks or months away from their home institutions, short-term exchanges offer similar opportunities while minimizing lost work productivity and costs associated with being away from homes and families. In addition, short-term exchanges offer cost-efficient training opportunities that do not require a significant amount of administrative oversight. Regardless of professional background, exchange programs are generally viewed quite favorably [3,7–9]. Nonetheless, data on such exchange experiences and their outcomes are limited, particularly for programs that focus on junior faculty exchanges between domestic institutions.

The purpose of the KL2 Visiting Scholars Program is to leverage the unique strengths of three regional CTSA hubs to promote cross-institutional training experiences for KL2 scholars. In particular, the program provides early-career scholars with opportunities to advance their program of research, establish cross-institutional collaborations, and expand their professional networks. The overarching goal of this manuscript is to present the structure and initial outcomes of the Visiting Scholars Program, encouraging other institutions to adopt and improve on our initial efforts in order to enhance career development opportunities for KL2 scholars.

## Materials and Methods

### KL2 Visiting Scholars Program History and Overview

The KL2 Visiting Scholars Program was initiated in 2016 by the University of Kentucky Center for Clinical and Translational Science (UK CCTS) in partnership with the Indiana Clinical and Translational Sciences Institute (IN CTSI) which includes Indiana University, Purdue University, and the University of Notre Dame. In 2018, the University of Cincinnati Center for Clinical and Translational Science and Training (UC CCTST) joined to expand the program partnership. The purpose of the Visiting Scholars Program has been to promote collaborative cross-CTSA training, leverage academic strengths at host CTSAs, and support the career development of our scholars through experiential training and the development of new partnerships. Scholars are competitively selected and have the opportunity to meet with faculty with similar research interests at the host institution. During their visit, scholars provide a formal presentation on their work, consult with senior research faculty, and visit with other KL2 scholars and CTSA program directors at the host institution. The first scholar exchange occurred in 2017. Since that time, 12 scholars have participated in the program and 6 more are scheduled to participate in 2021.

### Application and Selection Process

Each year, the KL2 Visiting Scholars Program announcement and application instructions are e-mailed to eligible scholars (Supplemental Material, Appendix A). Each participating CTSA KL2 Director sends the announcement to its own scholars encouraging them to apply by a specified date that is agreed upon by the participating institutions. Interested scholars respond to their home CTSA KL2 Director indicating their intent to apply, followed by formal application submission (Table 1).

**Table 1.** Application Materials

Item	Description
1	Paragraph summarizing the scholar’s program of research
2	List of CTSA institution(s) the scholar would like to visit
3	Rank-ordered list of faculty researchers the scholar would like to meet, including a brief explanation of how each researcher’s work potentially informs their own
4	Scholar’s NIH biosketch and full curriculum vitae

In advance of a selection/planning meeting, each partnering CTSA compiles application materials for their submitting scholars and distributes that information to the group for review. During the meeting, representatives from each participating CTSA review individual applications and select exchanges based on two criteria. First, there needs to be good networking opportunities for the candidate based on their area of research and identified faculty researchers. Second, efforts are made to ensure equal distribution of scholars across the participating institutions. Generally, each CTSA institution hosts two visiting scholars each year (one in the spring; one in fall). Applicants in their second year of training are given priority consideration. The summer and winter months are more difficult to schedule exchange experiences due to holidays, vacations, and in winter, possible inclement weather for traveling.

### Visiting Scholar Itinerary

Once selected, KL2 scholars are notified by their host CTSA KL2 Director, with the host CTSA taking responsibility for developing the visiting scholar’s itinerary. The host institution contacts the faculty members identified by the scholar (as part of the application process), as well as additional faculty identified by host CTSA leadership that may further enhance the scholar’s experience. Identified faculty receive a letter that provides an overview of the Visiting Scholars program, a copy of the visiting scholar’s NIH biosketch and CV, and an invitation to meet with the scholar (Supplemental Material, Appendix B). The host CTSA also contacts the scholar to request potential dates for their visit and then develops and schedules an itinerary. Typically, a visiting scholar will spend one full day visiting the host institution. See Table 2 for a typical itinerary.

**Table 2.** Example Visiting Scholar Itinerary

Schedule	Event
Day 1 (Evening)	Dinner with senior faculty and/or KL2 leadership the evening before their visit
Day 2 (Morning)	Breakfast with research faculty member and/or KL2 scholars from host institution
Day 2	Research presentation during Grand Rounds, a professional working group, or a school or department “work-in-progress” group consistent with the scholars’ area of research
Day 2	Individual meetings with several senior research faculty
Day 2 (Noon)	Lunch with research faculty members and/or KL2 scholars from host institution
Day 2	Tours or visits to labs and/or clinical areas relevant to the scholars’ research
Day 2	Exit interview with the host institution’s CTSA leadership

Once a final visit is confirmed, a formal invitation with a final itinerary is sent to the visiting scholar by the host institution’s CTSA KL2 Director (Supplemental Material, Appendix C), calendar invitations are sent to all participants, and plans for marketing the scholar’s presentation are finalized. The visiting scholar is provided with hotel recommendations, parking directions and passes, a campus map, contact information for key personnel during the visit, and any other assistance to make their visit a positive experience. The cost of meals is managed by the host institution, while travel costs for the visit are managed by the scholar’s home institution.

### Program Evaluation

Following their visit, scholars were asked to complete a brief *post-visit survey* about their exchange experience, with results used to make program improvements. The survey included seven items with a 5-point Likert-type rating scale response option (strongly agree to strongly disagree) (Table 3; Supplemental Material, Appendix E) and two open-ended items. Open response items included: (1) What were the things you found most useful regarding this Visiting Scholar Program? and (2) What were the things that could be improved regarding this Visiting Scholar Program?

**Table 3.** Post Visit Survey

Question	Response				
	Strongly Agree	Agree	Not Sure	Disagree	Strongly Disagree
Giving a formal presentation/talk during my visit was a valuable experience	10	2	0	0	0
Meetings I had with individual faculty or others during my visit were helpful	11	1	0	0	0
I wish my visit at the institution had been longer (i.e., more days)	2	3	4	3	0
The itinerary and other arrangements for my visit were well-organized	10	2	0	0	0
I have met at least 1 person I am likely to contact in the future	6	5	1	0	0
The visit was valuable in terms of my research or career development	8	3	1	0	0
I would recommend this visiting KL2 Scholar program to other KL2 trainees	11	0	1	0	0

To evaluate longer-term outcomes and impact of the KL2 Visiting Scholar Program, scholars were invited to complete an *outcomes survey* in June 2020. The survey was administered and completed using REDCap, and included items to identify ongoing professional interactions resulting from the exchange and types of outcomes resulting from those interactions (Table 4; Supplemental Material, Appendix F). The Institutional Review Boards (IRB) at each participating institution provided independent confirmation that these quality assurance/quality improvement evaluations did not meet the federal definition of research.

**Table 4.** Outcomes Survey

Questions	Response	
	Yes	No
Subsequent interaction(s) with any individual(s) that you met during the exchange visit	10	1
Plan to submit a federal grant with an individual that I met during the exchange visit	1	10
Plan to submit a foundation, industry, or other non-federal grant with an individual that I met	1	10
Written and submitted a federal grant with an individual that I met	1	10
Written and submitted a foundation, industry, or other non-federal grant with an individual that I met	1	10
Awarded a federal grant with an individual that I met	1	10
Awarded a foundation, industry, or other non-federal grant with an individual that I met	0	10
Plan to submit a manuscript with an individual that I met	2	9
Plan to submit a conference abstract or presentation with an individual I met	1	10
Co-authored a published manuscript with an individual I met	1	10
Co-authored a conference abstract or presentation with an individual I met	0	10
Follow-up communication with an individual(s) you met during the exchange visit?	10	1

## Results

Since the program began, a combined 60 scholars across the 3 CTSA institutions have been eligible to participate (50% physician-scientists, 65% female, and 18% underrepresented persons). To date, 20 scholars have applied to participate in the program, 12 scholars have been accepted and completed the program (no drop-outs to date), and 6 scholars are currently scheduled to complete the program during the 2020–2021 academic year (Table 5). Including those scheduled to participate, 9 (50%) will have been physician-scientists, 14 (78%) female, and 2 (11%) underrepresented persons. During the first year of the program, four scholars exchanged between UK CCTS and IN CTSI. The program was expanded in the second year to include the UC CCTST with eight scholars exchanging.

**Table 5.** Participant Demographics

Credentials	Exchanges		Gender		URP	CTS Research Type			
	Completed	Scheduled	Male	Female		T1	T2	T3	T4
PhD	6	1	2	5	0	2	5	0	0
PhD/RN	1	1	0	2	0	0	2	0	0
MD	1	3	1	3	2	2	1	0	0
MD/Dual Degree	4	1	1	4	0	2	2	1	1

URP, Underrepresented Person

Following the completion of the visiting scholar exchange, all participants received and completed the *post-visit survey* (*n* = 12; Table 3). All scholars agreed or strongly agreed that giving a formal presentation on their work and meetings with faculty at the host institution was beneficial and that their itinerary and visit were well organized. In addition, all but one scholar agreed or strongly agreed that the visit was valuable in terms of their research and/or career development, that they had identified at least one person to contact in the future, and that they would recommend the program to other KL2 scholars. The one item where scholars had varying opinions was with regard to the amount of time allotted for their visit, with nearly half of the scholars indicating the visit should be longer.

The outcomes survey (Table 4) was designed to assess longer-term program outcomes and was completed by 11 of the 12 visiting scholars. The timing of survey completion was 3-year post-visit for four scholars, and 2-year post-visit for seven scholars. A majority of scholars (10 of 11), responding to the long-term program outcomes survey, stated that they had subsequent interactions with the individuals they met with during their visit. To date, two scholars (18%) reported having submitted a collaborative extramural grant application, with one of those extramural grants funded. These same two scholars also reported plans to submit additional extramural grant applications. In addition, three scholars (27%) have plans to submit either a collaborative manuscript or an abstract with a faculty member they met at their host institution. Finally, one participant has published a manuscript with a faculty member they met with during their exchange and has established an ongoing collaboration with that faculty member.

Open-ended survey items for both the post-visit and outcomes surveys provide additional insights into the visiting scholar experience as well as ways to improve the program. Scholars felt the visit provided them with an opportunity to obtain outside feedback from experts in their area of research, as well as expanding their knowledge with regard to new ideas for how to approach their research questions. One scholar stated that “…the most useful element of the visit was visiting with potential collaborators and receiving feedback on work to date, identifying concrete opportunities for collaboration, and determining immediate next steps.” Overwhelming, the scholars’ open-ended comments highlighted opportunities to expand their network, such as “to identify new mentors” and “new experts in their field.” Scholars were tasked with identifying faculty members to meet with at the host institution. Several scholars identified this as an area for program improvement, stating that “they had very narrowly focused their selections and would have benefited more by working with the host institution to broaden their choices” and “I could have done a better job of identifying additional faculty outside of” their own discipline.

## Discussion

By leveraging the academic strengths of the University of Kentucky Center for Clinical and Translational Science (UK CCTS), the Indiana Clinical and Translational Sciences Institute, and the University of Cincinnati Center for Clinical and Translational Science and Training, the Visiting Scholar Program was successful in promoting the career development of KL2 scholars from all three institutions. Among 60 scholars who were eligible to participate, 20 (33.3%) applied for participation, and 18 (30%) were selected. Two applicants were not selected for program participation because the proposed networking opportunities were not strong. Program participants were representative of the broader population of eligible participants in terms of professional discipline, with female representation higher (78% vs. 65%) and underrepresented persons lower (11% vs. 18%; Table 5).

Based on evaluations completed immediately after program completion, all participants reported that both the opportunities to give an invited presentation and to participate in the Visiting Scholar Program were beneficial to career development, and a majority (11 of 12) reported that the program supported research development and was effective in expanding their collaborative networks (Table 3). More specifically, participants shared that the opportunity to engage in scholarly dialogue and critique with a new group of research experts helped them to refine and improve the rigor of their own work. Furthermore, the majority (11 of 12) of program graduates indicated they would recommend participation in the Visiting Scholar Program to other KL2 scholars.

For longer-term outcomes, a majority (10 of 11) of participants reported subsequent contact with investigators they met during their visit, including two collaborative grant submissions (one funded) and one collaborative manuscript submission 2- to 3-year post-program completion, as well as future plans for collaborative grant and manuscript submissions (Table 4). Given that only two scholars reported joint grant submissions and publications, we looked for potential reasons that might explain this level of outcome. One common factor identified was that these two scholars knew one or more investigators at the host institution prior to their visit. As a result, the Visiting Scholar Program served to strengthen those relationships and helped facilitate new connections beyond each scholars’ initial points of contact. For other scholars without a prior point of contact, the visit supported the formation of new connections and an additional program structures may be needed to encourage, support, and sustain new collaborations.

The overall impact of the program may be underreported, as 2- to 3-year intervals may not be adequate for collaborations to mature and translate into measurable outcomes such as joint grant submissions and publications. We recommend that outcomes be assessed at least annually (and perhaps at 6-month intervals) for at least 5 years following completion of the Visiting Scholar Program to fully capture the impact of the program on career development. We also recommend the addition of interviews to obtain more robust feedback and overcome the limitations of open-ended survey items [10]. While the long-term outcomes reported are modest, ongoing communication between scholars and faculty at the host institutions is ongoing for 10 of 11 visiting scholars, so it is possible that additional collaborative grants, manuscripts, and presentations will emerge over time.

Program improvements and modifications have occurred based on informal meetings with participating scholars and program directors. For example, based on informal feedback received from the first cohort of KL2 Visiting Scholars in 2018, we created a funding opportunity to promote and accelerate new research collaborations between visiting scholars and faculty at the host CTSA. To be eligible, the proposed multi-PI projects needed to involve collaboration between KL2 scholars who participated in the program and a faculty member at the host institution. Multi-PIs were required to have a full-time faculty appointment at their own CTSA institution, and scholars must have successfully completed their KL2 program at the time of pilot award funding. Funding was provided by the CTSAs of the collaborating multi-PIs, with funds spent at the CTSA that contributed funding (i.e., funds could not be transferred between institutions). Budget requests could not exceed $25,000 per CTSA ($50,000 total for direct costs only). Interested collaborators were asked to submit a joint letter of intent and biosketches for initial review. Upon invitation, the Multi-PIs were invited to submit a full proposal for a standard NIH-type study section review (see Supplemental Material, Appendix D: Funding Announcement for details).

The pilot grant was offered only one time and has been challenging to sustain. These challenges include partner institutions being on varying funding cycles, and the commitment of funds for the support of the pilot program was a concern at the end of a funding cycle when institutions were applying for continuation funding. Identifying additional sources of support for the pilot grant program could help to cover commitments during the times in which institutions are competing for renewed funding. The pilot grant program may be especially important in improving long-term outcomes for visiting scholar participants as it provides a more immediate mechanism to encourage and sustain newly developed cross-institutional parternships [11].

The cost of the Visiting Scholar Program is an important consideration related to program expansion. The cost of this Visiting Scholar Program has been modest, in large part due to regional collaboration which limits travel cost. The host institution covers the cost of three meals – dinner on the night prior to the Visiting Scholar Program (typically including the visiting scholar and KL2 leadership at the host institution), and breakfast and lunch for the scholar. Some host institutions scheduled the scholar presentations to occur during the lunch hour and provided food for individuals attending the invited presentation. The scholars’ CTSA covered other travel costs (mileage, hotel expenses for one night) that were manageable, given the proximity between CTSA institutions (200 miles separate UK and IU, 85 miles separate UK and UC, and 130 miles separate IU and UC).

There are modest administrative costs associated with managing the program and developing itineraries for visiting scholars, which are subsumed within the CTSA and KL2 program budgets. Finally, there are costs to the scholars in time spent in preparing program applications and preparation for the invited presentation and program. The program is highly scalable, with costs of expanding the program associated primarily with increased travel costs to the scholars. Opportunities for hosting the Visiting Scholar Program using virtual tools are also possible, which would significantly reduce travel costs and could increase institutional efficiency by providing guidance on program support. We have provided appendix materials to reduce the cost of program development by other CTSA’s or national programs supported virtually.

Several opportunities for programmatic improvement are apparent. For instance, we currently rely heavily on scholar mentors and KL2 program leadership to prepare visiting scholars to perform effectively and professionally in their roles as visiting scholars. The opportunity to provide training for effective engagement in visiting scholar invitations to both optimize performance and career development through the training of all KL2 scholars is a future opportunity. For example, leveraging the communicating science program at the Indiana CTSI to assist visiting scholars with their presentations, offer constructive criticism, or provide cross-institutional workshops [12]. Several scholars indicated that it would have been helpful to receive more input from the host institution in identifying potential faculty to meet during their visit. In this case, leveraging existing research development teams and advisory groups to help identify a more diverse group of senior scientists would help ensure young scholars form stronger, more diverse interdisciplinary collaborations [13].

The visiting scholars program is in the early stages of development and as a previously mentioned assessment of outcomes can be improved through an extended evaluation period and the addition of post-visit interviews. We anticipate that improvements to the program, including increased scholar support in the areas of communicating science and building networks, along with sustained availability of pilot grants will expand the number of visiting scholars who report sustained interdisciplinary collaborations and related outcomes.

In summary, the KL2 Visiting Scholar Program is a cost- and time-efficient program for enhancing KL2 scholar career development by leveraging the academic strengths of CTSAs to promote research development and expand professional collaborative networks. Resources developed to support the integrated UK CCTS, IN CTSI, and UC CCTST programs provided with this report should expedite the development of similar regional program development as well as national virtual visiting scholar opportunities.

## Supplementary material

For supplementary material accompanying this paper visit https://doi.org/10.1017/cts.2020.564. click here to view supplementary material

## References

[1] National Center for Advancing Translational Sciences. CTSA Program Hubs [Internet], 2020 [cited Aug 24, 2020]. (https://ncats.nih.gov/ctsa/about/hubs#:~:text=Program%20Funding%20Information-,CTSA%20Program%20Hubs,discovery%20into%20improved%20patient%20care)

[2] National Center for Advancing Translational Scineces. Supporting the Clinical and Translational Science Workforce [Internet], 2020, [cited Aug 24, 2020]. (https://ncats.nih.gov/files/NCATS_CTSA_Program_Training%20and%20Development_1.pdf)

[3] Van Schyndel JL , Koontz S , McPherson S , et al. Faculty support for a culture of scholarship of discovery: a literature review. Journal of Professional Nursing 2019; 35(6): 480–490. 31857059 10.1016/j.profnurs.2019.05.001

[4] McRae M , Zimmerman KM. Identifying Components of Success Within Health Sciences-Focused Mentoring Programs Through a Review of the Literature. American Journal of Pharmaceutical Education 2019; 83(1): 6976. PMC6418850 10.5688/ajpe6976 PMC6418850 30894774

[5] Gruppen LD , Frohna AZ , Anderson RM , Lowe KD. Faculty development for educational leadership and scholarship. Academic Medicine 2003; 78(2): 137–141. 12584091 10.1097/00001888-200302000-00007

[6] Thorndyke LE , Gusic ME , George JH , Quillen DA , Milner RJ. Empowering junior faculty: Penn State’s faculty development and mentoring program. Academic Medicine 2006; 81(7): 668–673. 16799296 10.1097/01.ACM.0000232424.88922.df

[7] McCarty RL , Fenn R , Gaster B , Weber W , Guiltinan J. Building bridges: qualitative assessment of a clinical faculty exchange between a naturopathic and an allopathic medical training program. Explore (NY) 2011; 7(4): 249–253. PMC3129540 21724159 10.1016/j.explore.2011.04.003 PMC3129540

[8] Cumbler E , Herzke C , Smalligan R , Glasheen JJ , O’Malley C , Pierce JR, Jr. Visiting professorship in hospital medicine: An innovative twist for a growing specialty. Journal of Hospital Medicine 2016; 11(10): 714–718. 27334568 10.1002/jhm.2625

[9] Ogdie A , Sparks JA , Angeles-Han ST , et al. Barriers and Facilitators of Mentoring for Trainees and Early Career Investigators in Rheumatology Research: Current State, Identification of Needs, and Road Map to an Inter-Institutional Adult Rheumatology Mentoring Program. Arthritis Care and Research 2018; 70(3): 445–453. PMC5700864 28544766 10.1002/acr.23286 PMC5700864

[10] Singla S , Adebayo OW , Shields K , Dorn L , Thiboutot D. Using exit interviews as one component of the KL2 program impact analysis method. Journal of Clinical and Translational Science 2020; 4(s1): 75.32313695

[11] Robinson GFWB , Schwartz LS , DiMeglio LA , Ahluwalia JS , Gabrilove JL . Understanding career success and its contributing factors for clinical and translational investigators. Academic Medicine 2016; 91(4): 570–582. 26509600 10.1097/ACM.0000000000000979 PMC4811729

[12] Rossing JP , Hoffmann-Longtin K . Chapter 12: Making sense of science: Applied improvisation for public communication of science, technology, and health. In: Dudeck TR , McClure C , eds. Applied Improvisation: Leading, Collaborating, and Creating beyond the Theatre. London, UK: Methuen Drama, 2018, pp. 245–266.

[13] Keller TE , Collier PJ , Blakeslee KL , McCracken K , Morris C. Early career mentoring for translational researchers: Mentee perspectives on challenges and issues. Teaching and Learning in Medicine 2014; 26(3): 211–216. 25010230 10.1080/10401334.2014.883983 PMC4114132

