# Supplementary material for: Visiting Scholars Program to enhance career development among early-career KL2 investigators in Clinical and Translational Science: Implications from a quality improvement assessment
Source: J Clin Transl Sci. 2020 Dec 14;5(1):e67. doi: 10.1017/cts.2020.564 (PMC8057478; doi:10.1017/cts.2020.564)
Supplement: Supplementary file 1 [file S2059866120005646sup001.pdf]

**Supplemental Material**

# Visiting Scholars Program to Enhance Career Development among Early Career KL2 Investigators in Clinical and Translational Science: Implications from a Quality Improvement Assessment

Sheri L. Robb, PhD[1]; Thomas H. Kelly, PhD[2,3]; Victoria L. King, PhD[3]; Jason Blackard, PhD[4]; Patricia C. McGuire, MS[5]

[1]School of Nursing, Indiana University, Indianapolis, IN, USA
[2]College of Nursing, University of Kentucky, Lexington, KY, USA
[3]College of Medicine, University of Kentucky, Lexington, KY, USA
[4]College of Medicine, University of Cincinnati, Cincinnati, OH, USA
[5]Indiana Clinical and Translational Sciences Institute, Indianapolis, IN, USA

Appendix A: Program Announcement E-mail with Application Instructions

Appendix B: Sample Invitation Letter to Senior Faculty

Appendix C: Sample Scholar Invitation Letter and Itinerary

Appendix D: Funding Announcement

Appendix E: Post-Visit Survey: KL2 Visiting Scholar Program Experience Survey

Appendix F: Outcomes Survey: KL2 Scholar Exchange – June 2020 Program/Visit Survey

## Appendix A: Sample Program Announcement E-Mail and Application Instructions

### Sample Letter

Dear [Insert KL2 Scholar(s) Name(s)],

We have an exciting opportunity for Scholars, called the ***CTSI KL2 Visiting Scholars Program***. This program will afford selected scholars the opportunity to travel, present, and meet with faculty/scholars at the **University of Kentucky Center for Clinical and Translational Science** and the **University of Cincinnati Center for Clinical and Translational Science and Training** in Fall 2020.

Program Highlights:

- One day, on campus visit as a CTSI KL2 Visiting Scholar (expenses covered)
- Present on your work as a visiting scholar
- Meet with several faculty who have shared research interests/expertise (you can request meetings with specific faculty)
- Receive feedback on your research program & career planning/trajectory

Benefits of Program Participation:

- Networking and exploring potential collaborations
- Feedback on research from an external group of research scholars
- Feedback on career planning & trajectory
- Excellent lines for your CV, including: Visiting Scholar Presentation (external, invited presentation) and Selection as a KL2 Visiting Scholar (Honor). Both items are good for your Promotion & Tenure portfolio.

The exchange experience would take place between September and November 2020, and we will work with scholars and host institutions to find a date that works best for everyone.

We are very pleased to bring this opportunity to you, and hope that you will consider exploring the benefits of this experience for your own program of research. If you are interested, please let us know of your **intent to apply by [insert date]**.*

Then we would ask that you read the attached instructions, and submit your application materials no later than **[insert date]***

**Please submit your intent and subsequent application materials to [insert name], with copy to [insert name].*

ADD Signature

# Application Instructions Attachment

## Application Instructions

Thank you for your interest in the CTSI KL2 Visiting Scholars Program, which affords KL2 scholars the opportunity to visit with research scholars from other CTSI hubs. In Fall 2020, there will be an opportunity for KL2 scholars to visit a partnering program. Partners include the University of Kentucky, the University of Cincinnati, and the Indiana CTSI (which include Indiana University (IU) Indianapolis, IU Bloomington, Purdue, and/or Notre Dame).

An important determinant for scholar selection is whether the host institution can develop a beneficial itinerary. While it is not possible to give any assurances at this point, each participating institution is invested in trying to accommodate any interests you might have in meeting with specific faculty. This information will be essential in determining whether the host institution can develop an appropriate and beneficial itinerary.

We would ask that you provide the following information:

- Paragraph summarizing your current program of research.
- Please provide a list of faculty at UK and/or UC that you would like to meet. Please force rank the list of faculty you would like to meet and provide a brief explanation of how each faculty's work would potentially inform your own.
- NIH Biosketch.
- Full CV.

In the pages that follow, you will find information from each host institution aimed at helping you search for faculty who may have shared research interests. We recognize that these strategies will not capture all opportunities, so we encourage you to explore and use other networking strategies as well.

**University of Kentucky (UK)**

Access to UK Faculty Information (funded investigators).

1. Access to a data base with a comprehensive list of UK faculty doing research in your area of interest requires a UK linkblue ID. To acquire a temporary linkblue ID please contact Victoria King, PhD. vicky.king@uky.edu  Requests for linkblue IDs take approximately 2 days

2. Your temporary linkblue ID will be emailed to you with instructions on activation

3. Following activation of your linkblue ID please use the following html to access the Keyword search to identify UK faculty that are involved in research in your area of interest.

https://ris.uky.edu/ospa/searchportal/Account/Login.aspx?ReturnUrl=%2fospa%2fsearchportal%2fDashboard%2fSearch.aspx

To find additional information:

1. Link to UK Homepage: http://www.uky.edu/UKHome/

2. Click the 'Search' tab in the upper right of the screen

3. Type in the faculty member's name.

**University of Cincinnati (UC)**

Researchers at UC: you can click on "Departments" and then "Divisions" and then the "faculty, staff and students" link.

https://med.uc.edu/faculty

Researchers at CCHMC: this is a list of all research divisions – clicking on a division name will take you to a page where you can click on a list of faculty and each should have their own webpage

https://www.cincinnatichildrens.org/research/divisions/view-all

College of Engineering

https://ceas.uc.edu/departments.html

College of Pharmacy

https://pharmacy.uc.edu/research

College of Nursing

https://nursing.uc.edu/research/researchers.html

**Indiana Clinical and Translational Sciences Institute**
**Collaborating Institutions/Faculty Directories**

**Indiana University School of Medicine**
http://medicine.iupui.edu/directory.aspx
Search the Indiana University School of Medicine Directory; select faculty and then division or search by a specific name or key word

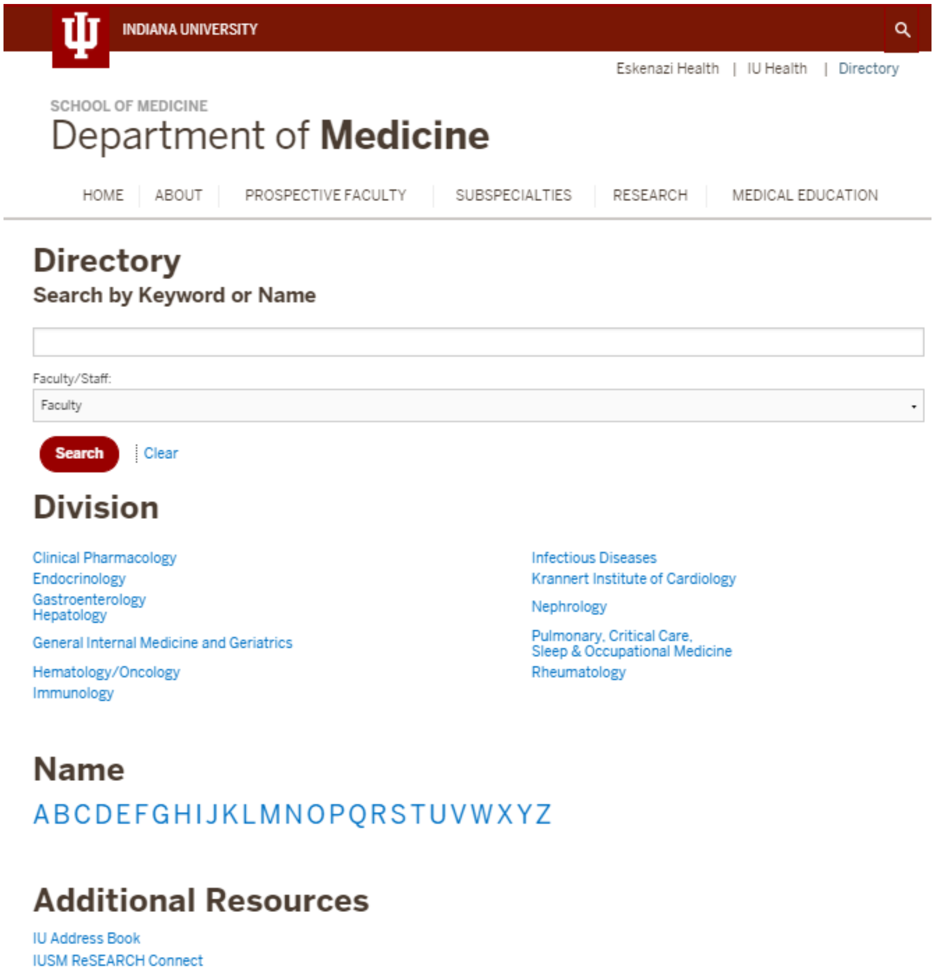

**Indiana University Purdue University Indianapolis (IUPUI) and Indiana University Bloomington (IUB)**
https://directory.iu.edu/
Search the IUPUI and/or IUB Directory; select either Indianapolis or Bloomington under campus and faculty under affiliation or search by specific name or department

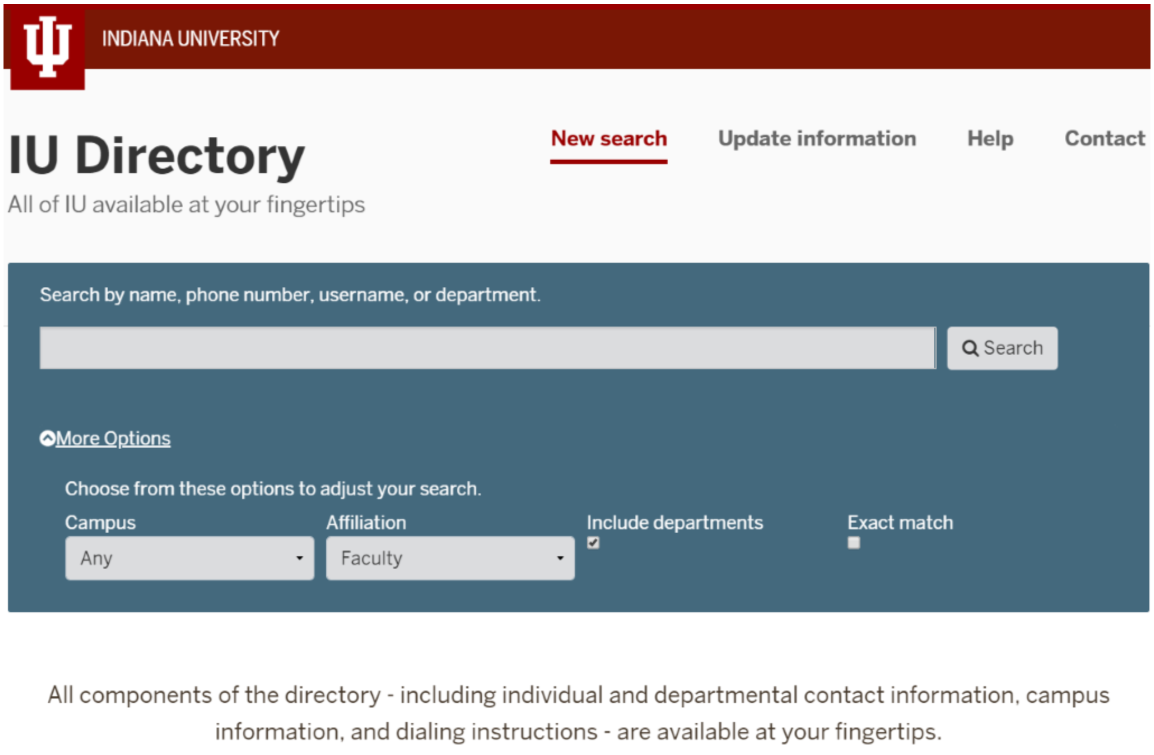


**Purdue University**
https://www.purdue.edu/directory/Advanced
Search the Purdue University Directory; under filters, select staff and West Lafayette, then filter by department and/or school

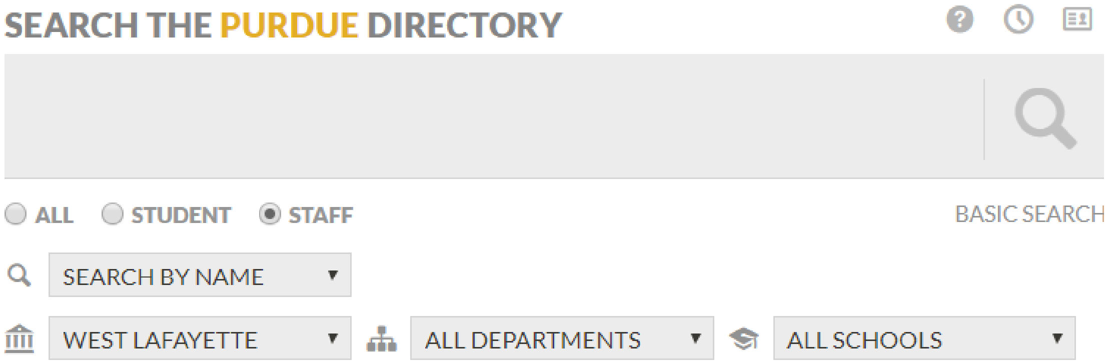

**University of Notre Dame**
https://apps.nd.edu/webdirectory/directory.cfm?specificity=begins&cn=ea&Submit=Submit
Search the University of Notre Dame Directory; under the “Available Searches” (left menu) type a department name (exp. Chemistry) under “Office and Department Directory”. After landing on a Department page select “Faculty” from the menu at the top of the page

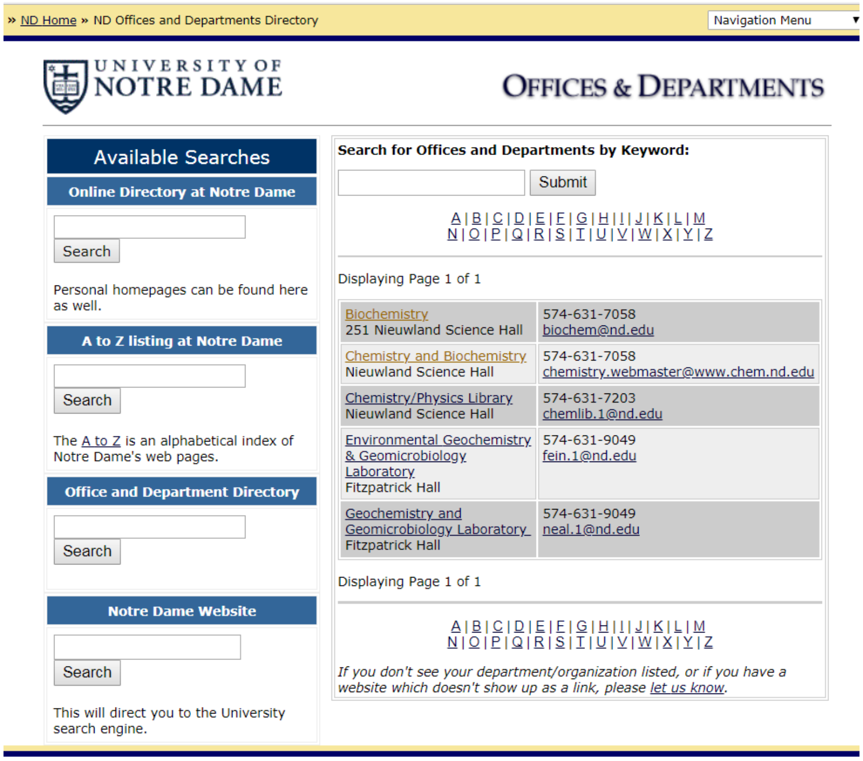


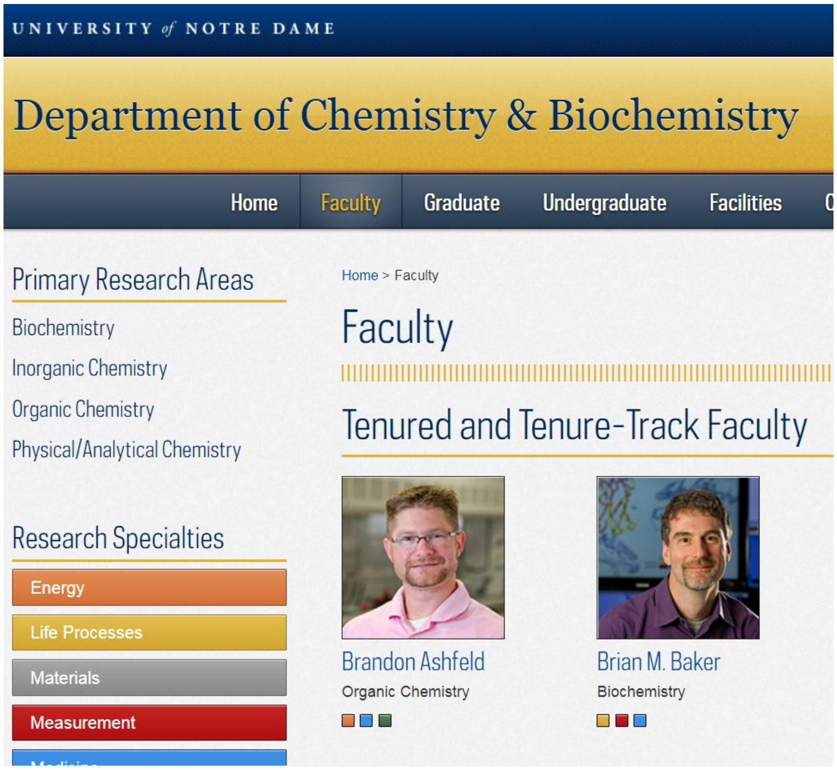

## Appendix B: Sample Invitation Letter to Senior Faculty

Dear [Insert Faculty Name and Credentials],

The KL2 Visiting Scholar Program is a partnership between the Indiana Clinical and Translational Sciences Institute (CTSI), the University of Kentucky Center for Clinical and Translational Sciences (CCTS), and the University of Cincinnati . The two institutions exchange KL2 scholars one or two times per year. The host institution coordinates the visit for the participating scholar.

Program highlights and benefits for the KL2 scholars include:

Program Highlights:

- One day, on campus visit as a CTSI KL2 Visiting Scholar (expenses covered)
- Present on work as a visiting scholar
- Meet with several faculty who have shared research interests/expertise
- Receive feedback on research program & career planning/trajectory

Benefits of Program Participation:

- Networking and exploring potential collaborations
- Feedback on research from an external group of research scholars
- Feedback on career planning & trajectory
- Excellent lines for their CV, including: Visiting Scholar Presentation (external, invited presentation) and Selection as a KL2 Visiting Scholar (Honor). Both items are good for their Promotion & Tenure portfolio.

We have a KL2 scholar from the University of Kentucky, [Full Name], [Credentials], who would like to visit the Indiana University School of Medicine campus next spring or fall [Year]. Dr. [Last Name] has expressed interest in meeting with you while she is here. If we can arrange a mutually convenient time, would you be willing to meet with Dr. [Last Name] for 45-60 minutes while she is in Indianapolis? I am attaching her CV and a sample visit itinerary. Previous visiting KL2 scholars have found the dedicated 1:1 time with senior faculty, who share similar research interests, to be most valuable. We are checking the availability and willingness of select faculty to meet with Dr. [Last Name] before we move forward with inviting her to visit. Will you meet with her?

Please let me know if you have any questions.

Add Signature

## Appendix C:  Sample Scholar Invitation Letter and Itinerary

### Sample Invitation Letter

[Month, Date, Year]

Dear Dr. [Last Name],

Congratulations on being selected to participate in the *[Year] KL2 Visiting Scholars Program.* We understand, in talking to Dr. Thomas Kelly at the University of Kentucky, Center for Clinical and Translational Science, that you are interested in visiting with faculty at the Indiana University School of Medicine, Indianapolis, IN. The visiting scholar program will provide you the opportunity to visit with faculty in our School of Medicine CTSI program who are aligned with your research, and present your work during Grand Rounds. You have indicated several faculty at our campus who are experts in your area of research. We have contacted these individuals and several have enthusiastically agreed to meet with you during your potential visit to our campus.

With this letter, we would like to invite you to visit our CTSI and the Indiana University School of Medicine campus in Indianapolis, Indiana [month, date, year]. If you are still interested in the KL2 Visiting Scholar program, please confirm your availability for those dates. I have attached a visit itinerary for your review.

Please do not hesitate to contact us with any questions or concerns, and we look forward to hosting you at the IU Indianapolis campus in [month].

Sincerely,

[insert signature]

Cc
[insert directors from cooperating sites]

# Sample Itinerary

**Itinerary for [Full Name], [Credentials], Visiting KL2 Scholar,**
**University of Kentucky, Center for Clinical and Translational Science**

**[Month, Date(s), Year] visiting Indiana University School of Medicine, Indianapolis**

**[Month, Date]**
***Travel to Indianapolis Visitor Parking*** *is available at the Health Information and Translational Sciences Building, 410 W. 10th Street, 46202, [insert name] will meet you in the lobby with a parking pass, plan to arrive a few minutes before 1:00 pm.*

**1:00 pm – 2:30 pm** [***Faculty Name]***, IUH Pathology Laboratory Bldg., 350 W. 11th St., then Joseph E. Walther Hall, 980 W. Walnut St., R3-C150, IU Simon Cancer Center Tissue Bank (*Insert name* will escort Dr. [KL2 Last Name])
**2:30 pm – 3:00 pm** ***Break and travel*** to Dr. [Name's] office (*Insert name* will escort Dr. [KL2 Last Name])
**3:00 pm – 4:00 pm** ***[Faculty Name]***, University Hospital, 550 N. University Blvd., 6th floor, Komen Tissue Bank, UH 6047 (*Insert name* will escort Dr. [KL2 Last Name])
**4:00 pm – 4:15 pm** ***Travel*** to Dr. [Name's] office (*Insert name* will escort Dr. [KL2 Last Name])
**4:15 pm – 4:45 pm** ***[Faculty Name]***, IU Simon Cancer Center, 3rd floor, Precision Genomics Center, off the Michigan St. elevators (*Insert name* will escort Dr. [KL2 Last Name])
**4:45 pm – 6:00 pm** ***Travel to hotel/dinner***
**6:00 pm – 8:00 pm** ***Dinner*** with ***insert name***, CTSI KL2 Director, ***insert name***, CTSI Director of Career Development, ***insert name***, CTSI Director of Mentoring Training, **XXX Restaurant**, [insert location]

**October 24**
***Travel to campus. Pay parking*** *is available at the Wilson Street Parking Garage, 811 Wilson St., 46202*

**9:15 am** ***Arrive at Cancer Research Institute 15 minutes prior to presentation***
**9:30 am – 10:30 am** ***Present at Cancer Center Educational Seminars***, Cancer Research Institute, Joseph E. Walther Hall, 980 W. Walnut St., R3, Room 303/305
**10:30 am – 11:30 am** ***Break and travel*** to *insert restaurant* (*insert name* will escort Dr. [KL2 Last Name])
**11:30 am – 12:30 pm** ***Lunch*** with ***Faculty Name,*** *title*, ***Faculty Name,*** *title*, and ***[Full Name]***, current KL2 Scholar, **insert restaurant name**, insert location.
**12:30 pm – 1:00 pm** ***Break and travel*** to Dr. [Name's] office (*Insert name* will escort Dr. [KL2 Last Name])
**1:00 pm – 2:00 pm** [***Faculty Name]***, Joseph E. Walther Hall, 980 W. Walnut St., R3, C130

***Depart to Lexington***

**Interactive Campus Map**
https://map.iu.edu/iupui/

**Point of contact during the visit will be**:
**Patty McGuire**, IN CTSI Education Program Manager, office) 317.278.2428 cell) XXX.XXX.XXXX

## Appendix D:  Funding Announcement

# KL2 Visiting Scholars Program - Pilot Grants

The Clinical and Translational Science Awards (CTSA) program at the National Institutes of Health (NIH) supports a national consortium of medical research institutions working to transform the way biomedical research is conducted. The program is designed to help accelerate the translation of laboratory discoveries into treatments for patients, train a new generation of clinical and translational researchers, and engage communities in clinical research efforts.

The purpose of the KL2 Visiting Scholars Program is to promote cross-CTSA training, enhance KL2 scholar career development, and enhance inter-CTSA research collaboration.  The associated Pilot Grants component of the program supports these objectives by funding innovative translational research projects that involve collaborations between KL2 scholars who have participated as visiting scholars and faculty at the host CTSA that they visited (multi-PIs).   Funding is provided by the CTSAs of the collaborating multi-PIs.  It is expected that funds will be spent at the CTSA that contributes the funding (funds will not be transferred between institutions). Budget requests must not exceed $25K per CTSA ($50K total for direct costs only).

**Eligibility** Proposed multi-PI projects must involve collaboration between KL2 scholars who have participated in the Visiting Scholar Program and a faculty member at the host CTSA institution that they visited. Multi-PIs must have a full-time faculty appointment at their own CTSA institutions. KL2 scholars must have successfully graduated from their KL2 program at the time of pilot award funding. Investigators are encouraged to contact their parent institution's Administrative Contact prior to LOI submission.

**Review Criteria**:  Letters of Intent and Full Applications will be reviewed by a study section composed of members of each participating CTSA.  Review criteria will include:

- Significance of the work
- Novelty/Innovation of the research idea
- Relevance of the proposed study to translational research
- Existence of a genuine collaborative multidisciplinary team in place at both CTSAs that is integral to the conduct of the research
- Evidence that the project could not be completed without the partnership between partner CTSAs
- Potential for the project to lead to future external funding or to a commercialization opportunity
- Soundness of the proposed methods
- Feasibility of accomplishing the stated project goals within a 12-month project period

**Application Deadline and Funding Cycle**:

- **Application Release Date: July 17, 2018**
- **LOI Deadline: September 3, 2018 5:00 pm EST**

Each collaborating Principal Investigator will work together on a single combined LOI. The Lead Institution's PI will submit the combined LOI using the following online submission Redcap database: https://redcap.uky.edu/redcap/surveys/?s=3TPJTADAL3

- **IRB/IACUC Submission Deadline: October 15, 2018**
- **Full Application Deadline: November 1, 2018 5:00 pm EST**
  Each collaborating Principal Investigator will work together on a single combined application. The Lead Institution's PI will submit the combined application within the following online submission Redcap database: https://redcap.uky.edu/redcap/surveys/?s=TPPKA93J9M
- **Notice of intent to fund: December 1, 2018**
- **IRB/IACUC Approval Deadline: December 5, 2018**
- **Submission to NIH for Prior Approval: December 10, 2018**
- **Funding Cycle: February 1, 2019 through January 31, 2020**

Applications that are late or do not adhere to the instructions may be administratively denied.

**LOI AND BIOSKETCH SUBMISSION GUIDELINES**

Designated representatives at collaborating CTSAs will review the submission and determine whether a full application will be encouraged.

<u>The LOI must be within a 2-page limit describing the following elements:</u>

- Research Objectives, Specific Aims
  - Provide concise, clear statements regarding anticipated outcomes of the proposed research and how it will add to existing knowledge or create value
- Background and Preliminary Data
- Study Design, Methodology and Anticipated Outcomes
- Description of Qualifications of KL2 Scholar and host CTSA Mentor
  - Additional members of the team (co-investigators) should be included and described (Approximately 1-2 paragraphs)
- Nature of the Collaboration
- Description of a plan on how these data will be used to submit a future grant proposal (priority will be given to applications with a more specific plan and timeline, eg. identification of an NIH program announcement and proposed time line for submission).
- Total Budget Request
  - KL2 CTSA $25K max
  - Host CTSA $25K max
  - Brief Budget Justification:

Biosketches of the Multi-PI's must also be attached (not included in the 2-page limit).

- ***Note that the expectation is that each CTSA's financial contribution to the pilot will be budgeted for activities at that location.***

***LOI submission link:*** ***https://redcap.uky.edu/redcap/surveys/?s=3TPJTADAL3***

**FULL RESEARCH PROTOCOL SUBMISSION PROCESS**

Once all CTSAs involved have approved the specific project, the investigators will write the full proposal and submit that to their own CTSAs for review. A standard NIH-type study section assessment will be organized involving reviewers from both institutions who will determine if they are supportive of going forward with the project.

Applicants are encouraged to review the instructions provided below carefully.

- Incomplete or incorrectly prepared applications will be returned without review.
- All applications exceeding the requested page limit will be rejected and not reviewed.
- The application margins must be no smaller than 0.5" at all points.
- Use an Arial, Helvetica, Palatino Linotype, or Georgia typeface, a black font color, and a font size of 11 points or larger. (A Symbol font may be used to insert Greek letters or special characters; the font size requirement still applies).
- Type density, including characters and spaces, must be no more than 15 characters per inch. Type may be no more than six lines per inch.
- EACH page should provide the applicant's name in the upper right-hand corner. The application should be numbered consecutively in the center bottom.

***Full Application submission link:*** https://redcap.uky.edu/redcap/surveys/?s=TPPKA93J9M

Applications should be assembled in the following order:

**I. Cover Page(s): (not included in the 6 page limit)**

1. Title of the Project and Total Amount Requested.
2. KL2 and host CTSA Multi-Principal Investigator Information:
   - Name
   - Degree(s)
   - Rank, Title (s)
   - College
   - Department /Division
   - Campus Address

**II. Detailed budget and budget justification in NIH format, direct cost only (not included in the 6 page limit)**

**Each multi-PI must submit a separate NIH budget proposal (limit $25K per institution)**

Allowable requests include:

- Equipment essential for the conduct of the study

- Data analysis costs
- Participant reimbursement costs
- Research assistant salary support
- Non-faculty personnel salary support
- Project specific specimen collection/analysis or testing
- Chemistry and biological lab supplies
- Purchase of cell lines, cultures reagents etc.
- Animal purchase and housing costs.
- Specimen collection/analysis or testing
- Participant reimbursement/recruitment costs

** Budgets must be pre-approved by their parent institution's Administrative Contact prior to submission.

Budget template can be downloaded here:
http://ccts.uky.edu/ccts/sites/default/files/RFA_doc/Page4_DetailedBudget.docx

## III. Abstract and Partnership development (not included in the 6 page limit).

Abstract: The abstract should provide a brief (not more than 250 word) summary of the project. Beneath the abstract, each of the key personnel and their departmental affiliation should be noted. The key personnel should minimally include multi-PIs from both CTSAs. Data analysis consultants (if included), collaborating investigators and others may be listed, if they will play a significant, active role in the conduct of the proposed work. Key personnel listed should provide a letter confirming their role (INCLUDE THESE LETTERS IN THE APPENDIX).

Explain how this partnership will provide new opportunities for the investigators, any development activities that will be conducted throughout the project, and how these activities will build a sustainable infrastructure for an ongoing partnership (not more than 250 words).

## IV. Body of the proposal: (limited to 6 pages)

The format of the application will follow NIH guidelines as outlined below.

### Specific Aims (limited to 1 page and included in the 6 pages of the body proposal)

State concisely the goals of the proposed research and summarize the expected outcome(s), including the impact that the results of the proposed research will exert on the research field(s) involved.

List succinctly the specific objectives of the research proposed, e.g., to test a stated hypothesis, create a novel design, solve a specific problem, challenge an existing paradigm or clinical practice, address a critical barrier to progress in the field, or develop new technology.

### Research Strategy

Organize the Research Strategy in the specified order and using the instructions provided below. Start each section with the appropriate section heading—Significance, Innovation, Approach. Cite published experimental details in the Research Strategy section and provide the full

reference in the Bibliography section. Given the length of the application, investigators should strive to provide a relevant, although not exhaustive bibliographic review (described below)

***(a) Significance***

- Explain the importance of the problem or critical barrier to progress in the field that the proposed project addresses.
- Explain how the proposed project will improve scientific knowledge, technical capability, and/or clinical practice in one or more broad fields.
- Describe how the concepts, methods, technologies, treatments, services, or preventative interventions that drive this field will be changed if the proposed aims are achieved.

***(b) Innovation***

- Explain how the application challenges and seeks to shift current research or clinical practice paradigms.
- Describe any novel theoretical concepts, approaches or methodologies, instrumentation or intervention(s) to be developed or used, and any advantage over existing methodologies, instrumentation or intervention(s).
- Explain any refinements, improvements, or new applications of theoretical concepts, approaches or methodologies, instrumentation or interventions.

***(c) Approach***

- Describe the overall strategy, methodology, and analyses to be used to accomplish the specific aims of the project. Include how the data will be collected, analyzed, and interpreted as well as any resource sharing plans as appropriate.
- Discuss potential problems, alternative strategies, and benchmarks for success anticipated to achieve the aims.
- If the project is in the early stages of development, describe any strategy to establish feasibility, and address the management of any high risk aspects of the proposed work.
- Clearly describe how each partner will be engaged in the development and/or implementation of the pilot study. (Applicable for partnership applications)

As applicable, also include the following information as part of the Research Strategy, keeping within the three sections listed above: Significance, Innovation, and Approach.

***(d) Preliminary Data***

Discuss the multi-PI's preliminary studies, data, and/or experience pertinent to this application. Preliminary data can be an essential part of a research grant application and help to establish the likelihood of success of the proposed project.

## V. References (not included in the 6 page limit).

Authors, year, title and journal information are expected for each citation. These **are not** included in the page limit and can be reported at the end of the body of the proposal.

## VI. Multi-PI Plan (not included in the 6 page limit).

This section should be no longer than 2 to 3 paragraphs. For instructions/examples of multi-PI plans, please see https://grants.nih.gov/grants/multi_pi/sample_leadership_plans.pdf.

## VII. Appendix (not included in the 6 page limit).

- LETTER FROM SUPERVISOR(s)/DEPARTMENT CHAIR(s)

  A letter signed by the immediate supervisor(s)/(e.g. Division Chief) and/or Department Chair that includes acknowledgement of their support for the project and providing assurance that sufficient protected time to complete the research will be available. No specific amount of protected time is required, but the review committee will consider the distribution of effort and other activities of the applicant.

- PIs and Co-PIs Biosketches in NIH format
- Protection of human-subjects section and animal assurances (if applicable)

*Applicants are prohibited from using the appendix to circumvent page limits in any section of the application for which a page limit applies.*

**REVIEW PROCESS & CRITERIA**

Full Applications will be sent to a minimum of two reviewers from each CTSA, with expertise in fields relevant to the science in the proposal. These reviewers will be asked to disclose any relationships to the grant applicant(s). Full proposals will be subject to a standard NIH-type study section assessment. The reviewers will provide written feedback addressing the merits of the protocol. All applications will be scored based upon the written reviews, relevance to the Priorities and Scope outlined above, and the overall relevance to the long-term goals of the CCTS. Applicants will be notified of the outcome.

The general criteria for review include:

- Significance of the work
- Novelty/Innovation of the research idea
- Relevance of the proposed study to translational research
- Existence of a genuine multidisciplinary team in place that is integral to the conduct of the research
- Evidence that the project could not be completed without the partnership between UK and the partner CTSA
- Potential for the project to lead to future external funding or to a commercialization opportunity
- Soundness of the proposed methods
- Feasibility of accomplishing the stated project goals within the 18 months project period

**IRB/IACUC GUIDELINES**

All applicants will be required to submit proof of IRB/IACUC submission (or proof of exempt status) at the time of Pilot Application Deadline. Applications without requisite IRB/IACUC submission prior to

October 15, 2018 will be administratively disqualified. IRB/IACUC approval will be required no later than December 5, 2018. Projects that do not have full IRB/IACUC approval by this date will not be considered for funding. **Please note all pilot IRB protocol titles must match the title of the CTSA pilot application.** Ongoing studies involving human subjects are eligible for pilot funding only if the project submitted for funding is a new unique project that has its own separate IRB submission and approval.

**AWARDEE RESPONSIBILITIES**

- Once your protocol is fully approved and funding awarded, you should contact your parent institution's Pilot Administrative Contact to schedule a working meeting with the CCTS units involved with your protocol.

- Successful applicants will be required to provide a written 6-month progress report followed by participation in a conference call with representatives from both CTSAs. A final written report describing project accomplishments must be submitted **within 60 days** of the project end date.

- The CTSAs are evaluated by the NIH on their effectiveness in stimulating new research findings and publications. **The following support acknowledgement should be included on all publications that result from support:**

***"This publication was supported by the National Center for Advancing Translational Sciences, National Institutes of Health, through Grants UL1TR002529 and UL1TR001998. The content is solely the responsibility of the authors and does not necessarily represent the official views of the NIH"***

# Appendix E: Post-Visit Survey

## KL2 Visiting Scholar Program Experience Survey

# KL2 Visiting Scholar Program Experience Survey

We appreciate you participating in the KL2 Visiting Scholar Program. We value your opinion about the things you liked about the experience and what aspects of the program might be improved in the future.

Please complete the survey below.

Thank you!

Enter your first and last name ______________________________

Select your institution or affiliate
- ◯ Indiana University
- ◯ University of Cincinnati
- ◯ University of Kentucky

## Experience

1.) Giving a formal presentation/talk during my visit was a valuable experience
- ◯ Strongly Disagree
- ◯ Disagree
- ◯ Not sure
- ◯ Agree
- ◯ Strongly Agree

2.) Meetings I had with individual faculty or others during my visit were helpful
- ◯ Strongly Disagree
- ◯ Disagree
- ◯ Not sure
- ◯ Agree
- ◯ Strongly Agree

3.) I wish my visit at the institution had been longer (i.e., more days)
- ◯ Strongly Disagree
- ◯ Disagree
- ◯ Not sure
- ◯ Agree
- ◯ Strongly Agree

4.) The itinerary and other arrangements for my visit were well-organized
- ◯ Strongly Disagree
- ◯ Disagree
- ◯ Not sure
- ◯ Agree
- ◯ Strongly Agree

5.) I have met at least 1 person I am likely to contact in the future
- ◯ Strongly Disagree
- ◯ Disagree
- ◯ Not sure
- ◯ Agree
- ◯ Strongly Agree

6.) The visit was valuable in terms of my research or career development
- ◯ Strongly Disagree
- ◯ Disagree
- ◯ Not sure
- ◯ Agree
- ◯ Strongly Agree

| | | |
|---|---|---|
| 7.) | I would recommend this visiting KL2 Scholar program to other KL2 trainees | ◯ Strongly Disagree<br>◯ Disagree<br>◯ Not sure<br>◯ Agree<br>◯ Strongly Agree |
| 8.) | What were the things you found most useful regarding this Visiting Scholar Program? | __________________________________ |
| 9.) | What were the things that could be improved regarding this Visiting Scholar Program? | __________________________________ |

# Appendix F:  Outcomes Survey

KL2 Scholar Exchange – June 2020 Program/Visit Survey

# KL2 Visiting Scholar Exchange - June 2020 program /visit survey

We are interested in learning about any outcomes or products from your KL2 Visiting Scholar exchange visit to Indiana University, the University of Kentucky, or the University of Cincinnati.

Please complete the brief survey below.

Thank you!

1) Enter your first and last name __________________________________

2) Select your institution or affiliate
- ☐ Indiana University
- ☐ University of Cincinnati
- ☐ University of Kentucky

3) Enter the month and year of your exchange visit __________________________________

4) Select the institution or affiliate that you visited as part of the exchange
- ☐ Indiana University
- ☐ University of Cincinnati
- ☐ University of Kentucky

**We are interested in learning about any outcomes or products from your exchange visit.**

5) Have you had any subsequent interaction(s) with any individual(s) that you met during the exchange visit?
- ◯ Yes
- ◯ No

6) Select all items that are applicable to your exchange visit

- ☐ I plan to submit a federal grant with an individual that I met with during the exchange visit
- ☐ I plan to submit a foundation, industry, or other non-federal grant with an individual that I met with during the exchange visit
- ☐ I have written and submitted a federal grant with an individual that I met with during the exchange visit
- ☐ I have written and submitted a foundation, industry, or other non-federal grant with an individual that I met with during the exchange visit
- ☐ I have been awarded a federal grant with an individual that I met with during the exchange visit
- ☐ I have been awarded a foundation, industry, or other non-federal grant with an individual that I met with during the exchange visit
- ☐ I plan to submit a manuscript with an individual that I met with during the exchange visit
- ☐ I plan to submit a conference abstract or presentation with an individual that I met with during the exchange visit
- ☐ I have co-authored a published manuscript with an individual that I met with during the exchange visit
- ☐ I have co-authored a conference abstract or presentation with an individual that I met with during the exchange visit

7) List / describe any other outcomes resulting from the exchange visit

__________________________________

8) Have you had any follow-up communication with an individual(s) you met during the exchange visit?

- ◯ Yes
- ◯ No

9) If you have had any follow-up communication with individuals met during your exchange visit, please list the type of communication (email, phone, in-person visit, etc) and frequency (one time only, weekly, monthly, etc).
If none: leave this field blank.

__________________________________________
